# Impact of Maternal Immunity on Congenital Cytomegalovirus Birth Prevalence and Infant Outcomes: A Systematic Review

**DOI:** 10.3390/vaccines7040129

**Published:** 2019-09-26

**Authors:** Tiziana Coppola, Jesse F. Mangold, Sarah Cantrell, Sallie R. Permar

**Affiliations:** 1 Department of Pediatrics, Duke University Hospital, Durham, NC 27701, USA; tiziana.coppola@duke.edu; 2 Duke HumanVaccine Institute, Durham, NC 27701, USA; jesse.mangold@duke.edu; 3 Medical Center Library & Archives, Duke University, Durham, NC 27701, USA; sarah.cantrell@duke.edu

**Keywords:** vertical transmission, hearing loss, reactivation, primary infection, congenital cytomegalovirus, maternal immunity

## Abstract

Congenital cytomegalovirus (cCMV) is the leading non-genetic cause of sensorineural hearing loss (SNHL), and efforts are geared towards prevention through vaccine development. Transmission rates following primary maternal infection occur at rates of 30–40%, however reported placental rates upon non-primary maternal infection is reported to be less than <4%. There is significant debate about whether this reduction in transmission rate is due to pre-existing maternal immunity, which could identify possible immunologic targets for vaccines. To address this question, we performed a systemic review of the literature using Preferred Reporting Items for Systematic Review and Analysis (PRISMA) guidelines. We identified cohort studies in high CMV seroprevalent (>80%) areas or in developing regions that examined a cohort of at least 50 infants for congenital CMV acquisition. We identified 19 articles that met criteria and were further categorized based on pre-conception serology, maternal seroprevalence, or previously known seroprevalence. Birth prevalence rates ranged from 0.4% to 6% (median 1.1%), with the studies reporting on clinical outcome (16/19 studies) noting the majority of infected infants as asymptomatic. We also utilized a recent study that differentiated primary maternal infections from chronic infections in a highly seropositive population to calculate a placental transmission rate in women with pre-existing immunity compared to that of no pre-existing immunity. This work confirms a low cCMV birth prevalence in highly seropositive populations, indicates via a calculated placental transmission rate that the CMV placental transmission rate is lower in non-primary infection than that of primary infection, and reveals gaps in data for further research aiming to identify targets for vaccine development.

## 1. Introduction

Congenital cytomegalovirus (cCMV) is the most common infectious cause of birth defects [1,2,3,4]. cCMV infection affects 5–7 of every 1000 live births in the United States and 0.5–2% of live births worldwide [5,6,7]. Each year, cCMV affects a total of 25,000–30,000 infants in the United States and more than 250,000 infants in India alone [8].

Approximately 85–90% of infants born with cCMV are asymptomatic, while only 10% of infants are symptomatic at birth [9,10]. Of those infants who demonstrate CMV-associated sequelae at birth, 40–60% will develop permanent sequelae including cerebral palsy, cognitive impairment, microcephaly and sensorineural hearing loss (SNHL) [7,10,11]. Those who are born asymptomatic are also at risk of developing sequelae; as 6–23% of asymptomatic infants eventually develop SNHL. Thus, cCMV is the leading non-genetic cause of SNHL [9,11]. Furthermore, as many as 5% of infants without recognizable CMV-associated sequelae at birth may develop microcephaly or neurodevelopmental deficits within the first year of life [10].

Placental transmission of CMV occurs in mothers with both prior natural immunity to CMV, termed ‘non-primary CMV infection’, and among seronegative mothers who contract CMV during pregnancy, termed ‘primary infection’ [2,5,12]. Congenital CMV infections occur in chronic CMV-infected women as a result from either reactivation of the virus from a previous infection or reinfection. The contribution of each of these potential routes of infection to non-primary cCMV incidence is not known. Recent studies show that seropositive mothers have high rates of reinfection with new virologic strains of CMV, ranging from 18–30% [13,14], suggesting that reinfection could be a major mode of non-primary cCMV infection. Birth prevalence of non-primary cCMV infection among infants born to all seropositive women is estimated to be 1%, yet placental transmission has been reported to be as high as 3.4% in women who have evidence of reinfection during pregnancy [8,15]. Seronegative mothers residing in areas of low CMV seroprevalence have low rates of maternal CMV acquisition ranging from 1–3% [1], yet placental CMV transmission rates in these cases of primary maternal CMV infection during pregnancy range from 30–50% [16,17,18]. In contrast, in areas of high CMV seroprevalence, seronegative mothers have high rates of CMV acquisition during pregnancy. For example, in a Brazilian cohort with a high CMV seroprevalence rate of 98.1%, the rate of CMV acquisition in seronegative mothers was reported to be 14%, 5/36 mothers [19]. Despite the high risk of maternal virus reactivation or reinfection, CMV placental transmission rates seem to be lower among mothers with non-primary infection compared to mothers with primary infection [6,20]. This data raises the question of the relative amount and nature of immunologic protection from prior CMV infection. Furthermore, it is unclear whether morbidity outcomes associated with cCMV infection are lower in the case of non-primary infection compared to primary infection. In a study by Ross et al. (2014), there were fewer cCMV-infected infants with bilateral hearing loss in the non-primary (23%) vs. the primary infected group (42%) and less progression of symptoms [13]. Furthermore, the risk of symptomatic cCMV infection of infants born in the setting of maternal non-primary infections is 1% versus 10–15% in primary infections [21]. For the purpose of this review, non-primary infection refers to maternal infections that have confirmed reinfection with CMV, while chronic CMV infection refers to a non-primary infection without confirmed reinfection status.

Current efforts for elimination of CMV infections are focused on vaccine development, which is regarded as a Level 1 priority by the National Academy of Medicine [22]. Further research must examine the impact of maternal immunity on CMV transmission and infant outcomes, as a deeper understanding of protective maternal immunity may guide future vaccine development. The goal of this systematic review is to characterize CMV birth prevalence (number of infants infected with CMV at birth/total number of infants studied), placental transmission rates (number of infants born with CMV/number of mothers who had primary or non-primary CMV infection in pregnancy) when reported, and infant outcomes in highly seropositive maternal populations, i.e., settings of pre-existing maternal CMV immunity. A previous review by Lanzieri et al. (2014) [23]) looked at birth prevalence of congenital CMV in developing countries, identifying rates of 0.6–6.1%. However, the calculation of birth prevalence from developing countries assumed all congenital infections were non-primary. This systematic review expands on the findings of Lanzieri et al (2014) [23] by grouping studies into tiers based on maternal serologic testing, allowing identification of the paucity of studies that assess mothers for non-primary versus primary infection. Improved understanding of cCMV transmission rates resulting from non-primary maternal infections will provide further evidence to study maternal immunity as a potential target for vaccine development.

## 2. Materials and Methods

We conducted a systematic review in concordance with the Preferred Reporting Items for Systematic Reviews and Meta-Analysis (PRISMA) guidelines [24]. Study selection was carried out by two authors who independently screened all available randomized clinical trial, cohort, and case-control studies cited within MEDLINE (via PubMed), EMBASE (via Elsevier), CINAHL (via EBSCO), Scopus (via Elsevier), and ClinicalTrials.gov from inception through 9 October 2018.

The search was conducted by a professional medical librarian and included a mix of keywords and subject headings representing cytomegalovirus, seroprevalence, and the pediatric population, respectively. Reproducible search strategies can be found in the Supplemental Materials Table S2. Editorials, letters, and comments were excluded, as were animal-only studies and studies not in English.

**Figure 1:**
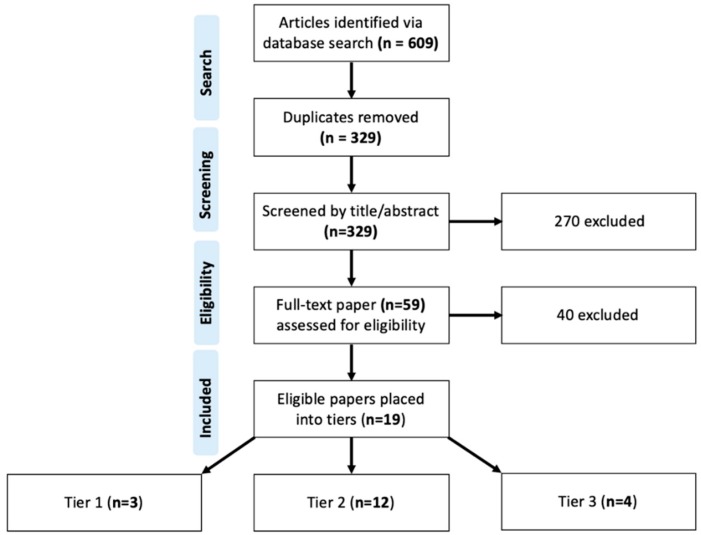
Systematic Review Flow Diagram.

Studies were excluded if they did not clearly meet inclusion criteria based on title and/or abstract review. All disagreements regarding eligibility were resolved through discussion between the reviewers or, if unable to be resolved, by adjudication by a third reviewer. Study selection is presented by flowchart as per PRISMA guidelines (Figure 1).

The search identified a total of (*n* = 609) references that were imported into an EndNote X9 (Clarivate Analytics) library and uploaded into Covidence, a systematic review screening tool. Duplicate citations (*n* = 280) were automatically identified and removed by Covidence. Two reviewers independently screened a total of (*n* = 329) references by title and abstract. Of those, (*n* = 270) references were deemed irrelevant and excluded. Tied references were broken by a third independent reviewer.

A total of (*n* = 59) articles were assessed in the full-text screening phase for eligibility given the following inclusion criteria: (1) employed a randomized control trial, cohort, or case-control study design; (2) studied a region of established high (>80%) maternal CMV seroprevalence or developing region; (3) screened at least (N = 50) neonates for CMV; and 4) utilized CMV DNA Polymerase Chain Reaction (PCR) of neonatal urine, saliva, blood, or dried blood spot (DBS) samples acquired within the first three weeks of life to confirm diagnosis of CMV infection. Studies that only utilized serological testing, such as detection of anti-CMV specific IgM antibodies via enzyme-linked immunosorbent assay (ELISA), to confirm diagnosis of congenital CMV infection were excluded on the basis that only 20–70% of infected infants present with anti-CMV specific IgM antibodies [25]. A further 40 studies were excluded based on study population, screening method for CMV, outcomes, study design and number of infants screened. A total of (*n* = 19) studies were ultimately included in the review. Data including birth prevalence rates (number of infants infected with CMV at birth/total number of infants studied), placental transmission rates (number of infants infected/number of mothers who had primary or non-primary CMV infection) symptoms, and symptom rates were extracted into an Excel spreadsheet (Table 1).

## 3. Results

The 19 studies that met our established criteria for studying cCMV transmission in highly seropositive populations were further broken down into three tiers. The Tier 1 studies (*n* = 3) addressed the chronicity of maternal CMV infection in study participants via assessment of IgG avidity and/or preconception CMV IgG immunity to differentiate primary from chronic maternal CMV infection. All Tier 1 studies completed IgG avidity testing on at least a portion of the pregnant subjects. The IgG avidity testing indicates whether an infection was primary or chronic based on the strength of IgG binding ratio with and without a dissociating reagent [46]. The Tier 2 studies (*n* = 12) assessed seroprevalence in mothers but did not specifically differentiate between primary or chronic CMV infections. Finally, Tier 3 studies (*n* = 4) were performed in highly CMV seroprevalent populations, however, did not formally test the seroprevalence of the mothers involved in the study. To correct for this, we utilized seroprevalence data from previous studies in similar regions [44,45]. Across the 19 studies in all three tiers, 386 infants were diagnosed with cCMV infections. The birth prevalence across all three tiers was 0.4 to 6%.

### 3.1. cCMV Birth Prevalence and Transmission Rates among Highly Seroprevalent Maternal Populations

#### 3.1.1. Tier 1 Studies

**Figure 2:**
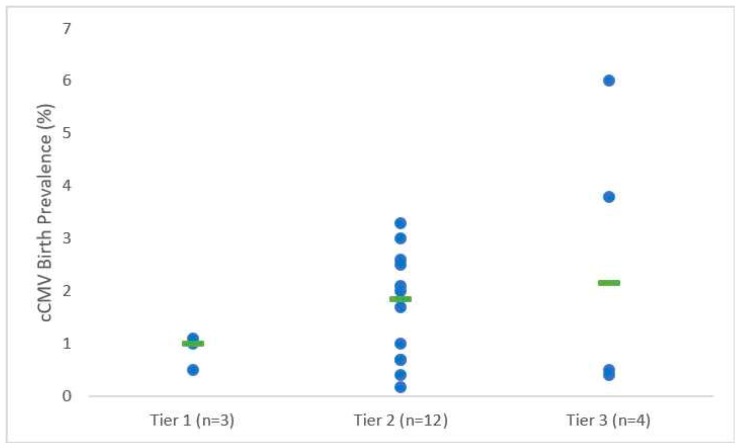
Congenital CMV birth prevalence rates within study tiers. Green bars indicate the median for each tier.

All Tier 1 studies utilized infant saliva or urine PCR with or without urine culture for confirmation of cCMV infection and diagnosed a majority (>89%) of pregnant subjects with chronic CMV infections based on high avidity IgG binding. In Tier 1 studies, cCMV birth prevalence rates in highly seropositive populations ranged from 0.5% to 1.1% with a median rate of 1% [19,27,47] (Table 1, Figure 2). All studies included IgG avidity testing to confirm chronic maternal infection on at least a subset of the pregnant subjects. Only one study, Mussi-Pinhata et al. in 2018, specifically examined cCMV rates in maternal primary versus chronic CMV infection within a highly seroprevalent maternal population. CMV serostatus was assessed in the first trimester, and the cCMV rates for both seronegative women and seropositive women, as well as the seronegative women with primary CMV acquisition during pregnancy were reported. Notably, there was a reduced cCMV birth prevalence among seropositive women (0.5%) versus preconception seronegative women (birth prevalence 2.8%) [19]. Moreover, there was a high rate of primary CMV acquisition during pregnancy in this population (14%, 5 of 36), demonstrating the frequent maternal CMV exposure in this population, and the observed placental transmission rate of 20% (1 in 5) is similar to what is expected in the setting of primary CMV acquisition. If the maternal CMV re-infection rate for the chronically infected mothers were assumed to be the same as that of the seronegative population (14% of seroimmune mothers potentially re-infected with CMV during pregnancy) producing a birth prevalence of 0.5%, then the calculated placental transmission rate for the chronically infected mothers would be 3.5%.

#### 3.1.2. Tier 2 Studies

Among the Tier 2 studies, 5 out of 12 utilized dried blood PCR for diagnosis of cCMV, whereas the remaining 7 studies utilized saliva or urine PCR and/or urine culture for diagnosis of cCMV. In these Tier 2 studies, the birth prevalence rate ranged from 0.4% to 3%, with a median of 1.85% [28,29,30,31,32,33,34,35,36,37,38,39]. These studies did not utilize avidity testing to indicate specifically if the maternal infection was recent. The study by Stagno et al. in 1982 had a limitation in its analysis of cCMV, as 79 samples were either contaminated or spilled, reducing the total number of samples (119 of 197, 60%), and possibly resulting in an over reported prevalence of 1.7% [36].

#### 3.1.3. Tier 3 Studies

All 4 studies in Tier 3 utilized urine and saliva PCR for diagnosis of cCMV, and 2 of 4 studies also utilized blood PCR as an alternative method of analyzing cCMV prevalence rates. In Mwaanza et al. four different methods were utilized to detect transmission, including PCR saliva, PCR urine, PCR sera, and IgM sera [42]. There was not significant overlap between the assays with only 3 of 15 samples (20%) testing positive in more than one domain. Among these Tier 3 studies, the birth prevalence rate ranged from 0.4% to 6% with a median rate of 2.15% [40,41,42,43].

### 3.2. Sequelae of cCMV Infection Among Infants Born to CMV Seropositive Mothers in Highly Seroprevalent Populations

In 16 of 19 studies in our systematic review that reported outcomes of the cCMV-infected infants, the overwhelming majority of cCMV-infected infants were asymptomatic. Of the 16 studies which included symptoms, the most commonly identified was hearing loss. In total, 5.7% of infants with cCMV infections (22/386) were reported to have hearing loss (7/16 studies reporting, Table 2). Abnormal brain imaging was found in 5.4% (14/257) of cCMV-infected infants screened with imaging (8/16 studies reporting, Table 2). Other common findings included jaundice in 4.4% (17/386) of cCMV-infected infants (5/16 studies reporting, Table 2), hepatosplenomegaly in 2.3% (9/386; 4/16 studies reporting, Table 2), and “small for gestational age” in 3.1% (12/386; 2/16 studies reporting, Table 2). Regarding motor and cognitive delays, there were only three studies that mentioned symptoms of cerebral palsy (1 patient in 1 study, Table 2), intellectual disability (1 patient in 1 study, Table 2), and hypertonia (1 patient in 1 study, Table 2), respectively, out of the 386 collectively identified cCMV-infected infants.

### 3.3. Hearing Loss among cCMV-Infected Infants Born to CMV Seropositive Mothers in Highly Seroprevalent Populations

In 37% (7/19) of studies, infants were assessed for hearing loss, a known common sequela of cCMV infection. In Tier 1 studies, hearing loss rates measured after birth ranged from 8.6–9.8%. Mussi-Pinhata et al. (2009) found that 8% (2/27) of infants born to mothers with chronic CMV infections had bilateral hearing loss at follow-up, although they were originally asymptomatic (Table 2) [27]. In the same study, one infant born to a mother with primary infection had late bilateral hearing loss at 24 months [27]. In Tier 2 studies, hearing loss rates ranged from 0–40% (Table 2). In one study by Barbi et al. (2006), hearing loss was transient and improved by future visits [31]. In another study by Dar et al. (2008), hearing loss was not present until four months in 11% (1/9) of patients and was unilateral in nature [38]. Only one Tier 3 study by Karimian et al. (2016) assessed hearing loss, reported at a rate of 12.5% (1/8) of patients (Table 2) [41].

### 3.4. Prematurity Among cCMV-Infected Infants Born to CMV Seropositive Mothers in Highly Seroprevalent Populations

Two studies identified whether neonates infected with cCMV were born premature and differentiated cCMV prevalence rates for pre-term versus term infants [29,37]. The studies differed on cCMV prevalence rates for the pre-term versus term neonates. In Yamamoto et al. (2001), cCMV was found in 2.1% (6/289) of preterm infants and 1.8% (3/163) of term infants, but the difference in birth prevalence between preterm and term infants was not statistically significant [29]. In this study, all cCMV infants that were clinically symptomatic were born prematurely. In Alkhawaja et al. (2012), cCMV was found in 1.9% (2/84) of preterm infants vs. 6.3% (1/16) of term infants [37], but symptoms were not tracked [29,37].

## 4. Discussion

Our systematic review identified 19 studies which evaluated CMV-seropositive mothers and the associated cCMV birth prevalence. Of those, 16 studies reported symptoms of affected infants. Overall, cCMV birth prevalence ranged from 0.4% to 6% [19,26,27,29,30,31,32,33,34,35,36,37,38,39,40,41,42,43]. Tier 1 studies, which differentiated primary versus chronic CMV maternal infections, had a median birth prevalence rate of 1%. Studies collectively showed that the overwhelming majority of cCMV-infected infants were asymptomatic. Of those infants with symptoms, hearing loss (5.7% of patients, 7/16 studies) followed by abnormal brain imaging (5.4% of patients, 8/16 studies) and jaundice (4.4% of patients, 5/16 studies) were the most common symptoms. Cognitive and motor delays were only reported in 3 cCMV-infected infants within 3 studies. There was insufficient information in this review to make conclusions regarding differential rates of cCMV infections amongst preterm and full term infants.

Lanzieri et al. (2014) conducted a systematic review evaluating birth prevalence of CMV in developing countries. In their work, eleven studies reported CMV maternal seroprevalence rates of 84–100% and CMV birth prevalence of 0.6% to 6.1% [23]. Our systematic review found similar birth prevalence, however, we were additionally able to report placental transmission rates for a study that distinguished chronic CMV infections from primary infections. The birth prevalence for this study demonstrated a lower cCMV birth prevalence in maternal chronic CMV infections compared with primary infections (0.5% vs. 2.8% respectively) [19]. The median birth prevalence for all three tiers of studies, including primary and chronic CMV infections, ranged from 1% to 2.1%. The highest median birth prevalence rate was 2.15% in Tier 3, among studies that did not specifically test maternal seroprevalence.

Mothers residing in areas of low maternal CMV seroprevalence acquire CMV during pregnancy at a low rate of 1–4% [21]. The placental transmission rate for primary CMV infection is very high at 30–50% [48]. Other studies show that transmission rates for primary infections can be as high as 78%, with the rate increasing with gestational age, as it can range from 30.1% transmission in the first trimester to 72.2% in the third trimester [49,50]. These high transmission rates in women who are acutely infected are in stark contrast to the low birth prevalence in seropositive populations found from our systematic review, which ranged from 0.4% to 6% [19,26,27,29,30,31,32,33,34,35,36,37,38,39,40,41,42,43], despite expected high CMV exposure rate in these populations. This range mirrors work by Fowler et al., (2003) in whichupon comparing primary versus non-primary maternal CMV infections they found that 3% of infants were infected by primary maternal infection versus 1% by non-primary maternal infection [34]. The only study within our systematic review that directly compared birth prevalence among primary and chronic maternal CMV infections, performed by Mussi-Pinhata et al., (2018) had similar results [12,19]. Of five pregnant women who seroconverted (14%, 5/36), only one gave birth to an infant with cCMV, resulting in a placental transmission rate of 20% [19]. Our calculated 3.5% placental transmission rate for reinfection of chronically infected mothers, which applies the acquisition rate from the seronegative mothers within the study cohort, is considerably lower than the 20% placental transmission rate for primary infected mothers in this study [19]. The cCMV birth prevalence and calculated transmission rates among seronegative women in a highly CMV seroprevalent population compared to that of their seropositive neighbors is revealing and may demonstrate the protective role of pre-existing maternal immunity.

The risk of early symptomatic cCMV due to primary infection is around 10–15%, but cCMV-infected infants who are initially asymptomatic have a 25% risk of developing sequelae by 24 months. In comparison, the risk of symptomatic cCMV due to non-primary infection is as low as 1%, and the risk of developing late sequelae is approximately 8% [21]. Findings from the studies included in our systematic review were not consistent with these prior findings, as some studies showed upwards of 40% of cCMV-infected infants being symptomatic for those infected following chronic CMV infections [32]. When we focused solely on Tier 1 studies that differentiated primary from chronic maternal infections, symptomatic cCMV-infected infants were as high as 10%. The symptomatic infants were born to mothers with chronic CMV infections. In the Mussi Pinhata et al. study in 2018 comparing primary vs. chronic CMV infections, none of the cCMV-infected infants were symptomatic in either primary or chronic group. Despite the high rates of symptoms for cCMV born to chronically infected mothers compared to previous studies, all studies showed that the majority (55–100%) of cCMV infected infants from chronic CMV infections were asymptomatic. It is unclear why the symptom rate is higher than expected from the prior literature, but may be due to small sample size, variable screening for sequelae of infection, and how long the infants were monitored for symptoms.

Overall, this review demonstrated that maternal immunity to CMV conferred protection as evidenced by median birth prevalence rates of 1.0–2.1% amongincluded studies. This low birth prevalence in a setting of high expected CMV exposure in a seropositive population suggests partial maternal immune protection against cCMV transmission. Current efforts for CMV prevention focus on vaccine development. Future studies must look at maternal T cells and antibody responses in transmitting and non-transmitting seropositive mothers in order to identify potential targets for vaccines to prevent congenital CMV. However, there are several limitations to our systematic review. First, the gold standard for identifying cCMV is viral culture of saliva or urine, which is not always collected in newborns. Instead, dried blood spots are more commonly collected. The utilization of PCR has decreased the length of time for detection. Urine PCR detection has sensitivities ranging from 93–100%, while saliva PCR has sensitivity of approximately 97% [47,51]. In contrast, dried blood PCR has lower sensitivity than that of saliva or urine PCR, making the likelihood of missing cases very high [52]. In our systematic review, 37% (7/19 studies) utilized dried blood PCR, however only 11% (2 of 19 studies) relied solely on that test for diagnosis [30,39]. The studies utilizing dried blood spots as an additional method also used urine, saliva or combination of both. Another limitation of our study is that 21% (4/19) of studies did not assess seroprevalence in the study population [40,41,42,43]. Instead, overall seroprevalence was inferred from similar populations within the same country for the purposes of this review. Importantly, none of the studies assessed for maternal reinfection, which does not provide a full assessment of non-primary infections and indicates the need for studies of placental transmission rates following established maternal re-infection. Another limitation considered is the potential underreporting of hearing loss due to limited infant follow up, as many infants may be diagnosed with hearing loss later in development despite being asymptomatic at birth [9]. Many of the studies in our review assessed hearing loss within the first few weeks of life with minimal long-term follow-up for SNHL, likely decreasing the identified rates of symptomatic SNHL in this patient population versus what was expected.

## 5. Conclusions

This study is one of the most comprehensive, structured systematic reviews demonstrating the overall low birth prevalence of cCMV and symptoms in highly seroprevalent populations. Importantly, we identified studies that differentiated primary from chronic maternal infections. Moreover, we highlight one study that reported lower birth prevalence for chronically-infected women than that of seronegative women within the same population, and when applying the infection rate in the seronegative women to the chronically infected women, indicates a lower transmission rate in chronically infected women. Our review reaffirms findings from previous studies and provides further evidence for a protective role of pre-existing maternal immunity against cCMV transmission. Further research should focus on studying placental transmission rates following maternal reinfection, as well as the immune responses in seropositive mothers that influence transmission rates to their infants.

## Figures and Tables

**Table 1:** Summary of study methods for assessing congenital cytomegalovirus (cCMV) birth prevalence in highly seroprevalent countries.

	Study	Region	Mothers (N)	Maternal Seroprevalence (%)	IgG Avidity Testing (n/N, %)			Testing Method	
					Low	High	Undetermined	Mothers	Infants
**Tier 1**	Mussi-Pinhata et al. (2018) [19]	Brazil	1952 ^A^	98.1	5/36 ^B^	1915/1952 (98.1)	N/A	Serum Ab	Saliva PCR
	Yamamoto et al. (2011) [26]	Brazil	452 ^C^	96.7	1/44 (2.2) ^D^	39/44 (88.6) ^D^	3/44 (6.8) ^D^	Serum Ab	Saliva or Urine PCR, Culture (Cx)
	Mussi-Pinhata et al. (2009) [27]	Brazil	7848 ^E^	95.7 ^F^	1/42 (2.4) ^D^	41/42 (97.6) ^D^	N/A	Serum Ab	Saliva/Urine PCR and Saliva/Urine Cx
**Tier 2**	Arellano-Galindo et al. (2014) [28]	Mexico	375 ^G^	97.3				Serum Ab	Dried Blood PCR, Saliva PCR, Cx
	Yamamoto et al. (2001) [29]	Brazil	452	95.7				Serum Ab	Urine PCR, Cx
	Wang S et al. (2017) [30]	China	5020 ^H^	96.2 ^F^				Dried Blood Ab	Dried Blood PCR, Saliva PCR
	Barbi et al. (2006) [31]	Italy	9032	80.0 ^F^				Serum Ab	Dried Blood PCR, Urine Cx
	Kaneko et al. (2013) [32]	Japan	1163	83.3				Serum Ab	Urine PCR
	Madrid et al. (2018) [33]	Mozambique	118 ^I^	100				Serum Ab	Dried Blood PCR
	Ziyaeyan et al. (2007) [34]	Iran	92	98.6				Serum Ab	Blood PCR
	Fowler et al. (2003) [35]	USA	2857	100				Serum Ab	Saliva Rapid Cx, Saliva/Urine Cx
	Stagno et al. (1982) [36]	Chile	197 ^J^	98				Serum Ab	Urine Cx
	Alkhawaja et al. (2012) [37]	Bahrain	83 ^K^	100				Serum Ab	Saliva/Urine PCR, Saliva/Urine Cx
	Dar et al. (2008) [38]	India	423	99				Serum Ab	Saliva PCR, Urine PCR
	Schlesinger et al. (2003) [39]	Israel	1939 ^L^	82.6				Dried Blood Ab	Urine PCR, Cx
**Tier 3**	Viswanathan et al. (2018) [40]	India	750	99				Serum Ab	Blood PCR, Saliva PCR, Urine PCR
	Karimian et al. (2016) [41]	Iran	Not reported	97.6 ^M^				Not tested	Urine PCR
	Mwaanza et al. (2014) [42]	Sub-Saharan Africa	395	87.8 ^N^				Not tested	Saliva PCR, Serum PCR, Urine PCR, Serum IgM
	Tshabalala et al. (2018) [43]	South Africa	302	87.8 ^N^				Not tested	Saliva PCR

^A^ 1952 mothers were tested for CMV seroprevalence. Thirty-six mothers were CMV-seronegative and had their infants included in study. However, among 1915 seropositive women, only 1685 infants were enrolled in cCMV screening; ^B^ Rate of CMV-seronegative women who became CMV-IgG positive during pregnancy; ^C^ Maternal seroprevalence based on Yamamoto et al. (2001) [29]. ^D^ Only a randomly selected subset of mothers was tested for IgG avidity. ^E^ 7848 mothers with 7656 singletons, 185 twins and 7 triplets. ^F^ Calculated based on randomly selected subset of maternal cohort. ^G^ 375 mothers were tested for CMV seroprevalence, however only 277 infants were enrolled in cCMV screening. ^H^ Unclear discrepancy between maternal and infant sample size. ^I^ 3 infant samples were deemed invalid for viral determination due to sample error. ^J^ 79 infant samples were contaminated and were excluded from analysis. ^K^ 83 mothers with 67 singletons, 15 twins and 1 triplet. ^L^ 2000 infants were included in the study, while maternal epidemiological data were available for only 1939 mothers. ^M^ Maternal seroprevalence based on Tabatabaee et al. (2009) [44]. ^N^ Maternal seroprevalence based on Bates et al. (2016) [45].

**Table 2:** Summary of selected studies cCMV birth prevalence and symptoms in highly seroprevalent countries.

	Study	cCMV Rate (n/N, %)		Asymptomatic Infants (n, %)	Symptomatic Infants (n, %)	Symptoms (n)	Hearing Loss Rate (Type; n/N, %)	
							At Birth	After Birth
**Tier 1**	Mussi-Pinhata et al. (2018) [19]	Low: 1/36 (2.8) ^A^	High: 8/1685 (0.47) ^A^	9 (100)	0 (0)	None	Not reported	Not reported
	Yamamoto et al. (2011) [26]	121/12195 (1.0)		109 (90.1)	12 (9.9)	Small for gestational age (8), Abnormal CT findings (3), Death (1)	Not reported	SNHL: 10/102 (9.8)
	Mussi-Pinhata et al. (2009) [27]	87/8047 (1.1) ^E^		80 (92.0)	7 (8.0)	Hepatosplenomegaly (4), Jaundice (4), Petechiae (4), Thrombocytopenia (4), Abnormal CT findings (3), Elevated CSF protein (3), Elevated alanine/aspartate aminotransferase (3), Purpura (2), Seizures (1)	Not reported	SNHL: 3 bilateral, 2 unilateral; 5/58 (8.6)
**Tier 2**	Arellano-Galindo et al. (2014) [28]	7/277 (2.5) ^G^		7 (100)	0 (0)	None	Not reported	Not reported
	Yamamoto et al. (2001) [29]	9/452 (2.0)		5 (55.6)	4 (44.4)	Hepatosplenomegaly (3), Intrauterine growth restriction (2), Hypertonia (1), Microcephaly (1), Petechiae (1), Subcortical and periventricular hypodensity of white matter (1)	Not reported	Not reported
	Wang S et al. (2017) [30]	75/10933 (0.69) ^H^		71 (94.7)	4 (5.3)	Hearing loss (2), Jaundice (2)	Bilateral: 2/75 (2.7)	Not reported
	Barbi et al. (2006) [31]	16/9032 (0.18)		14 (87.5)	2 (12.5)	Elevated direct bilirubin (2), Elevated alanine aminotransferase (1), Cerebral calcifications (1)	Transient unilateral: 2/16 (12.5)	Not reported
	Kaneko et al. (2013) [32]	5/1163 (0.43)		3 (60)	2 (40)	Cerebral palsy (1), Intellectual disability (1)	Bilateral: 2/5 (40.0)	Not reported
	Madrid et al. (2018) [33]	3/115 (2.6) ^I^		3 (100)	0 (0)	None	Not reported	Not reported
	Ziyaeyan et al. (2007) [34]	3/92 (3.3)		3 (100)	0 (0)	None	Not reported	Not reported
	Fowler et al. (2003) [35]	29/2857 (1.0)		Not reported	Not reported	Not reported	Not reported	Not reported
	Stagno et al. (1982) [36]	2/118 (1.7) ^J^		2 (100)	0 (0)	None	Not reported	Not reported
	Alkhawaja et al. (2012) [37]	3/100 (3.0) ^K^		Not reported	Not reported	Not reported	Not reported	Not reported
	Dar et al. (2008) [38]	9/423 (2.1)		8 (88.9)	1 (11.1)	Hepatosplenomegaly (1)	0/9 (0)	Unilateral: 1/9 (11.1)
	Schlesinger et al. (2003) [39]	14/2000 (0.70) ^L^		12 (85.7)	2 (14.3)	Hepatosplenomegaly (1), Microcephaly (1), Petechiae (1), Periventricular calcifications (1), Small for gestational age (1), Thrombocytopenia (1), Transient mild hepatitis (1)	0/10 (0)	Not reported
**Tier 3**	Viswanathan et al. (2018) [40]	3/750 (0.40)		2 (66.7)	1 (33.3)	Lethargy (1), Low birth weight (1), Preterm (1), Respiratory distress (1), Sepsis (1)	Not reported	Not reported
	Karimian et al. (2016) [41]	8/1617 (0.49)		5 (62.5)	3 (37.5)	Jaundice (3), Respiratory distress (3), Suspected sepsis (3)	Mild: 1/8 (12.5)	Not reported
	Mwaanza et al. (2014) [42]	15/395 (3.8)		9 (60.0)	6 (40.0)	Jaundice (6), Death (4), Petechiae (1), Respiratory distress (1)	Not reported	Not reported
	Tshabalala et al. (2018) [43]	18/302 (6.0)		Not reported	Not reported	Not reported	Not reported	Not reported

^A^ 1952 mothers were tested for CMV seroprevalence. Thirty-six mothers were CMV-seronegative and had their infants included in study. However, among 1915 seropositive women, only 1685 infants were enrolled in cCMV screening; ^B^ Rate of CMV-seronegative women who became CMV-IgG positive during pregnancy; ^C^ Maternal seroprevalence based on Yamamoto et al. (2001) [29]. ^D^ Only a randomly selected subset of mothers was tested for IgG avidity. ^E^ 7848 mothers with 7656 singletons, 185 twins and 7 triplets. ^F^ Calculated based on randomly selected subset of maternal cohort. ^G^ 375 mothers were tested for CMV seroprevalence, however only 277 infants were enrolled in cCMV screening. ^H^ Unclear discrepancy between maternal and infant sample size. ^I^ Three infant samples were deemed invalid for viral determination due to sample error. ^J^ 79 infant samples were contaminated and were excluded from analysis. ^K^ Eighty-three mothers with 67 singletons, 15 twins and 1 triplet. ^L^ 2000 infants were included in the study, while maternal epidemiological data were available for only 1939 mothers. ^M^ Maternal seroprevalence based on Tabatabaee et al. (2009) [44]. ^N^ Maternal seroprevalence based on Bates et al. (2016) [45].

## Supplementary Materials

The following are available online at https://www.mdpi.com/2076-393X/7/4/129/s1, File S1: Search strategies for cCMV transmission in seropositive maternal populations.

## References

[1] Permar S.R., Schleiss M.R., Plotkin S.A. (2018). Advancing Our Understanding of Protective Maternal Immunity as a Guide for Development of Vaccines to Reduce Congenital Cytomegalovirus Infections. J. Virol..

[2] Wang C., Zhang X., Bialek S., Cannon M.J. (2011). Attribution of congenital cytomegalovirus infection to primary versus non-primary maternal infection. Clin. Infect. Dis.

[3] Giannattasio A., Di Costanzo P., De Matteis A., Milite P., De Martino D., Bucci L., Augurio M.R., Bravaccio C., Ferrara T., Capasso L. (2017). Outcomes of congenital cytomegalovirus disease following maternal primary and non-primary infection. J. Clin. Virol..

[4] Van Zuylen W.J., Hamilton S.T., Naing Z., Hall B., Shand A., Rawlinson W.D. (2014). Congenital cytomegalovirus infection: Clinical presentation, epidemiology, diagnosis and prevention. Obstet Med..

[5] Bialas K.M., Permar S.R. (2016). The March towards a Vaccine for Congenital CMV: Rationale and Models. PLoS Pathog..

[6] Kenneson A., Cannon M.J. (2007). Review and meta-analysis of the epidemiology of congenital cytomegalovirus (CMV) infection. Rev. Med. Virol..

[7] Fowler K.B., Boppana S.B. (2018). Congenital cytomegalovirus infection. Semin. Perinatol..

[8] Britt W.J. (2017). Congenital Human Cytomegalovirus Infection and the Engima of Maternal Immunity. J. Virol..

[9] Marsico C., Kimberlin D.W. (2017). Congenital Cytomegalovirus infection: Advances and challenges in diagnosis, prevention and treatment. Ital. J. Pediatr..

[10] Boppana S.B., Ross S.A., Fowler K.B. (2013). Congenital Cytomegalovirus Infection: Clinical Outcome. Clin. Infect. Dis..

[11] Schleiss M.R., Permar S.R., Plotkin S.A. (2017). Progress toward Development of a Vaccine against Congenital Cytomegalovirus Infection. Clin. Vaccine Immunol..

[12] Fowler K.B., Stagno S., Pass R.F., Britt W.J., Boll T.J., Alford C.A. (1992). The Outcome of Congenital Cytomegalovirus Infection in Relation to Maternal Antibody Status. N. Engl. J. Med..

[13] Ross S.A., Arora N., Novak Z., Fowler K.B., Britt W.J., Boppana S.B. (2010). Cytomegalovirus reinfections in healthy seroimmune women. J. Infect. Dis..

[14] Yamamoto A.Y., Mussi-Pinhata M.M., Boppana S.B., Novak Z., Wagatsuma V.M., Oliveira P.D.F., Duarte G., Britt W.J. (2010). Human cytomegalovirus reinfection is associated with intrauterine transmission in a highly cytomegalovirus-immune maternal population. Am. J. Obstet. Gynecol..

[15] Simonazzi G., Curti A., Cervi F., Gabrielli L., Contoli M., Capretti M.G., Rizzo N., Guerra B., Farina A., Lazzarotto T. (2018). Perinatal Outcomes of Non-Primary Maternal Cytomegalovirus Infection: A 15-Year Experience. Fetal Diagn. Ther..

[16] Davis N.L., King C.C., Kourtis A.P. (2017). Cytomegalovirus infection in pregnancy. Birth Defects Res..

[17] Johnson J., Anderson B. (2013). Cytomegalovirus: Should We Screen Pregnant Women for Primary Infection?. Am. J. Perinatol..

[18] Stagno S., Pass R.F., Cloud G., Britt W.J., Henderson R.E., Walton P.D., Veren D.A., Page F., Alford C.A. (1986). Primary cytomegalovirus infection in pregnancy. Incidence, transmission to fetus, and clinical outcome. JAMA.

[19] Mussi-Pinhata M.M., Yamamoto A.Y., Aragon D.C., Duarte G., Fowler K.B., Boppana S., Britt W.J. (2018). Seroconversion for Cytomegalovirus Infection During Pregnancy and Fetal Infection in a Highly Seropositive Population: “The BraCHS Study”. J. Infect. Dis..

[20] Leruez-Ville M., Magny J.F., Couderc S., Pichon C., Parodi M., Bussieres L., Guilleminot T., Ghout I., Ville Y. (2017). Risk factors for congenital cytomegalovirus infection following primary and non-primary maternal infection: A prospective neonatal screening study using polymerase chain reaction in saliva. Clin. Infect. Dis..

[21] Hughes B.L., Gyamfi-Bannerman C. (2016). Diagnosis and antenatal management of congenital cytomegalovirus infection. Am. J. Obstet. Gynecol..

[22] Stratton K.R., Durch J.S., Lawrence R.S., Institute of Medicine (2000). Executive Summary. Vaccines for the 21st Century: A Tool for Decisionmaking.

[23] Lanzieri T.M., Dollard S.C., Bialek S.R., Grosse S.D. (2014). Systematic review of the birth prevalence of congenital cytomegalovirus infection in developing countries. Int. J. Infect. Dis..

[24] Liberati A., Altman D.G., Tetzlaff J., Mulrow C., Gøtzsche P.C., Ioannidis J.P.A., Clarke M., Devereaux P., Kleijnen J., Moher D. (2009). The PRISMA statement for reporting systematic reviews and meta-analyses of studies that evaluate healthcare interventions: Explanation and elaboration. BMJ.

[25] Revello M.G., Gerna G. (2002). Diagnosis and Management of Human Cytomegalovirus Infection in the Mother, Fetus, and Newborn Infant. Clin. Microbiol. Rev..

[26] Yamamoto A.Y., Mussi-Pinhata M.M., Isaac M.D.L., Amaral F.R., Carvalheiro C.G., Aragon D.C., Manfredi A.K.D.S., Boppana S.B., Britt W.J. (2011). Congenital cytomegalovirus infection as a cause of sensorineural hearing loss in a highly immune population. Pediatr. Infect. Dis. J..

[27] Mussi-Pinhata M.M., Yamamoto A.Y., Brito R.M.M., Isaac M.D.L., Oliveira P.F.D.C.E., Boppana S., Britt W.J., Britto R.M.M. (2009). Birth prevalence and natural history of congenital cytomegalovirus infection in a highly seroimmune population. Clin. Infect. Dis..

[28] Arellano-Galindo J., Villanueva-García D., Cruz-Ramirez J.L., Yalaupari-Mejìa J.P., Uribe-Gutiérrez G., Velazquez-Guadarrama N., Nava-Frias M., Munoz-Hernández O., Mejía-Arangure J.M. (2014). Detection and gB genotyping of CMV in Mexican preterm infants in the context of maternal seropositivity. J. Infect. Dev. Ctries.

[29] Mussi-Pinhata M.M., Pinto P.C.G., Jorge S.M., Yamamoto A.Y., Figueiredo L.T.M. (2001). Congenital cytomegalovirus infection in preterm and full-term newborn infants from a population with a high seroprevalence rate. Pediatr. Infect. Dis. J..

[30] Wang S., Wang T., Zhang W., Liu X., Wang X., Wang H., He X., Zhang S., Xu S., Yu Y. (2017). Cohort study on maternal cytomegalovirus seroprevalence and prevalence and clinical manifestations of congenital infection in China. Medicine.

[31] Barbi M., Binda S., Caroppo S., Calvario A., Germinario C.A., Bozzi A., Tanzi M.L., Veronesi L., Mura I., Piana A. (2006). Multicity Italian Study of Congenital Cytomegalovirus Infection. Pediatr. Infect. Dis. J..

[32] Kaneko M., Sameshima H., Minematsu T., Kusumoto K., Yamauchi A., Ikenoue T. (2013). Maternal IgG avidity, IgM and ultrasound abnormalities: Combined method to detect congenital cytomegalovirus infection with sequelae. J. Perinatol..

[33] Madrid L., Varo R., Maculuve S., Nhampossa T., Muñoz-Almagro C., Calderón E.J., Esteva C., Carrilho C., Ismail M., Vieites B. (2018). Congenital cytomegalovirus, parvovirus and enterovirus infection in Mozambican newborns at birth: A cross-sectional survey. PLoS ONE.

[34] Ziyaeyan M., Alborzi A., Abbasian A., Kalani M., Moravej A., Nasiri J., Amiri A., Hashemi N., Sefiddashti F. (2007). Detection of HCMV DNA in placenta, amniotic fluid and fetuses of seropositive women by nested PCR. Eur. J. Pediatr..

[35] Fowler K.B., Stagno S., Pas R.F. (2003). Maternal Immunity and Prevention of Congenital Cytomegalovirus Infection. Obstet. Gynecol. Surv..

[36] Stagno S., Dworsky M.E., Torres J., Mesa T., Hirsh T. (1982). Prevalence and importance of congenital cytomegalovirus infection in three different populations. J. Pediatr..

[37] Alkhawaja S., Ismaeel A., Botta G., Senok A.C. (2012). The prevalence of congenital and perinatal cytomegalovirus infections among newborns of seropositive mothers. J. Infect. Dev. Ctries..

[38] Dar L., Pati S.K., Patro A.R.K., Deorari A.K., Rai S., Kant S., Broor S., Fowler K.B., Britt W.J., Boppana S.B. (2008). Congenital cytomegalovirus infection in a highly seropositive semi-urban population in India. Pediatr. Infect. Dis. J..

[39] Schlesinger Y., Halle D., Eidelman A., Reich D., Dayan D., Rudensky B., Raveh D., Branski D., Kaplan M., Shefer V. (2003). Urine polymerase chain reaction as a screening tool for the detection of congenital cytomegalovirus infection. Arch. Dis. Child. Fetal Neonatal Ed..

[40] Viswanathan R., Bafna S., Mergu R., Deshpande G., Gunjikar R., Gaikwad S., Mullick J. (2018). Direct Saliva Real Time Polymerase Chain Reaction Assay Shows Low Birth Prevalence of Congenital CMV Infection in Urban Western India. Pediatr. Infect. Dis. J..

[41] Karimian P., Yaghini O., Azadani H.N., Mohammadizadeh M., Arabzadeh S.A.M., Adibi A., Rahimi H. (2016). Prevalence, Characteristics, and One-Year Follow-Up of Congenital Cytomegalovirus Infection in Isfahan City, Iran. Interdiscip. Perspect. Infect. Dis..

[42] Mwaanza N., Chilukutu L., Tembo J., Kabwe M., Musonda K., Kapasa M., Chabala C., Sinyangwe S., Mwaba P., Zumla A. (2014). High rates of congenital cytomegalovirus infection linked with maternal HIV infection among neonatal admissions at a large referral center in sub-Saharan Africa. Clin. Infect. Dis..

[43] Tshabalala D., Newman H., Businge C., Mabunda S.A., Kemp W., Beja P. (2018). Prevalence and determinants of congenital cytomegalovirus infection at a rural South African central hospital in the Eastern Cape. S. Afr. J. Infect. Dis..

[44] Tabatabaee M., Tayyebi D. (2009). Seroepidemiologic study of human cytomegalovirus in pregnant women in Valiasr Hospital of Kazeroon, Fars, Iran. J. Matern. Neonatal Med..

[45] Bates M., Brantsaeter A.B. (2016). Human Cytomeglovirus (CMV) in Africa: A neglected but important pathogen. J. Virus Erad..

[46] Prince H.E., Lapé-Nixon M. (2014). Role of Cytomegalovirus (CMV) IgG Avidity Testing in Diagnosing Primary CMV Infection during Pregnancy. Clin. Vaccine Immunol..

[47] Ross S.A., Ahmed A., Palmer A.L., Michaels M.G., Sanchez P.J., Bernstein D.I., Tolan R.W., Novak Z., Chowdhury N., Fowler K.B. (2014). Detection of Congenital Cytomegalovirus Infection by Real-Time Polymerase Chain Reaction Analysis of Saliva or Urine Specimens. J. Infect. Dis..

[48] Ornoy A., Diavcitrin O. (2006). Fetal effects of primary and secondary cytomegalovirus infection in pregnancy. Reprod. Toxicol..

[49] Yinon Y., Farine D., Yudin M.H. (2010). Screening, Diagnosis, and Management of Cytomegalovirus Infection in Pregnancy. Obstet. Gynecol. Surv..

[50] Enders G., Daiminger A., Bader U., Exler S., Enders M. (2011). Intrauterine transmission and clinical outcome of 248 pregnancies with primary cytomegalovirus infection in relation to gestational age. J. Clin. Virol..

[51] Boppana S.B., Ross S.A., Shimamura M., Palmer A.L., Ahmed A., Michaels M.G., Sánchez P.J., Bernstein D.I., Tolan R.W., Novak Z. (2011). Saliva polymerase-chain-reaction assay for cytomegalovirus screening in newborns. N. Engl. J. Med..

[52] Boppana S.B., Ross S.A., Novak Z., Shimamura M., Tolan R.W., Palmer A.L., Ahmed A., Michaels M.G., Sánchez P.J., Bernstein D.I. (2010). Dried blood spot real-time polymerase chain reaction assays to screen newborns for congenital cytomegalovirus infection. JAMA.

