# Supplementary material for: Impact of Maternal Immunity on Congenital Cytomegalovirus Birth Prevalence and Infant Outcomes: A Systematic Review
Source: Vaccines (Basel). 2019 Sep 26;7(4):129. doi: 10.3390/vaccines7040129 (PMC6963523; doi:10.3390/vaccines7040129)
Supplement: Supplementary file 1 [file vaccines-07-00129-s001.pdf]

vaccines

MDPI

Review

Impact of Maternal Immunity on Congenital Cytomegalovirus Birth Prevalence and Infant Outcomes: A Systematic Review

Tiziana Coppola, Jesse F. Mangold, Sarah Cantrell and Sallie R. Permar

Supplementary Materials: Search strategies for cCMV transmission in seropositive maternal populations.

MEDLINE (via PubMed)

Search date: 10/9/2018

#1	"cytomegalovirus infections"[Mesh] OR "cytomegalovirus vaccines"[Mesh] OR "cytomegalovirus"[Mesh] OR "cytomegalovirus-specific hyperimmune globulin"[supplementary concept] OR cytomegalovirus[tw] OR cytomegalic[tw] OR "cmv infection"[tw] OR "cmv infections"[tw] OR "ccmv infection"[tw] OR "ccmv infections"[tw] OR "cmv disease"[tw] OR "ccmv disease"[tw]	48,102
#2	"Seroconversion"[Mesh] OR seroconversion[tw] OR seroprevalence[tw] OR "sero-positive"[tw] OR seropositive[tw] OR seropositivity[tw]	76,463
#3	“congenital”[sh] OR congenital[tw]	327,361
#4	#1 AND #2 AND #3	308
#5	("Child"[Mesh] OR "Infant"[Mesh] OR "Adolescent"[Mesh] OR "Pediatrics"[Mesh] OR Infant[tw] OR infants[tw] OR infancy[tw] OR newborn[tw] OR newborns[tw] OR neonatal[tw] OR neonate[tw] OR neonates[tw] OR baby[tw] OR babies[tw] OR preterm[tw] OR prematurity[tw] OR toddler[tw] OR toddlers[tw] OR boy[tw] OR boys[tw] OR boyhood[tw] OR girl[tw] OR girls[tw] OR girlhood[tw] OR kid[tw] OR kids[tw] OR child[tw] OR children[tw] OR schoolchild[tw] OR school child[tw] OR school age[tw] OR adolescent[tw] OR adolescents[tw] OR adolescence[tw] OR juvenile[tw] OR juveniles[tw] OR youth[tw] OR youths[tw] OR teen[tw] OR teens[tw] OR teenager[tw] OR teenagers[tw] OR pubescent[tw] OR pubescence[tw] OR prepubescent[tw] OR prepubescence[tw] OR pediatric[tw] OR pediatrics[tw] OR paediatric[tw] OR paediatrics[tw]) NOT ("Adult"[Mesh] NOT ("Adolescent"[Mesh] OR "Child"[Mesh] OR "Infant"[Mesh]))	3,929,935
#6	#4 AND #5	239
#7	#6 NOT (Editorial[pt] OR Letter[pt] OR Comment[pt])	237
#8	#7 NOT (animals[mh] NOT humans[mh])	235
#9	#8 AND English[lang]	218

EMBASE

Search date: 10/9/2018

#1	'cytomegalovirus infection'/exp OR 'Cytomegalovirus'/exp OR 'Cytomegalovirus antibody'/exp OR 'Cytomegalovirus vaccine'/exp OR cytomegalovirus:ab,ti,kw OR cytomegalic:ab,ti,kw OR 'cmv infection':ab,ti,kw OR 'cmv infections':ab,ti,kw OR 'ccmv infection':ab,ti,kw OR 'ccmv infections':ab,ti,kw OR 'cmv disease':ab,ti,kw OR 'ccmv disease':ab,ti,kw	73,988

#2	'seroconversion'/exp OR seroconversion:ab,ti,kw OR seroprevalence:ab,ti,kw OR 'sero-positive':ab,ti,kw OR seropositive:ab,ti,kw OR seropositivity:ab,ti,kw	80,604
#3	congenital:ab,ti,kw	330,027
#4	#1 AND #2 AND #3	433
#5	('child'/exp OR 'adolescent'/exp OR 'pediatrics'/exp OR [child]/lim OR [infant]/lim OR [adolescent]/lim OR child:ab,kw,ti OR infant:ab,kw,ti OR infants:ab,ti,kw OR infancy:ab,ti,kw OR infantile:ab,ti,kw OR newborn:ab,ti,kw OR newborns:ab,ti,kw OR neonate:ab,ti,kw OR neonates:ab,ti,kw OR neonatal:ab,ti,kw OR baby:ab,ti,kw OR babies:ab,ti,kw OR preterm:ab,ti,kw OR premature:ab,ti,kw OR prematurity:ab,ti,kw OR toddler:ab,ti,kw OR toddlers:ab,ti OR children:ab,ti,kw OR childhood:ab,ti,kw OR kid:ab,ti,kw OR kids:ab,ti,kw OR schoolchildren:ab,ti,kw OR schoolchild:ab,ti,kw OR preadolescent:ab,ti,kw OR adolescent:ab,ti,kw OR adolescence:ab,ti,kw OR youth:ab,ti,kw OR youths:ab,ti,kw OR teen:ab,ti,kw OR teenager:ab,ti,kw OR teens:ab,ti,kw OR teenagers:ab,ti,kw OR teenage:ab,ti,kw OR teenaged:ab,ti,kw) NOT (([young adult]/lim OR [adult]/lim OR [middle aged]/lim OR [aged]/lim OR [very elderly]/lim) NOT ([embryo]/lim OR [fetus]/lim OR [newborn]/lim OR [infant]/lim OR [child]/lim OR [adolescent]/lim))	4,126,897
#6	#4 AND #5	313
#7	#6 NOT ('editorial'/exp OR 'letter'/exp OR 'note'/exp OR [conference abstract]/lim)	269
#8	#7 AND [humans]/lim	249
#9	#8 AND [english]/lim	222

CINAHL

Search date: 10/9/2018

#1	(MH "Cytomegalovirus Infections+") OR (MH "Cytomegaloviruses") OR "cytomegalovirus infection" OR TI(cytomegalovirus OR cytomegalic OR "cmv infection" OR "cmv infections" OR "ccmv infection" OR "ccmv infections" OR "cmv disease" OR "ccmv disease") OR AB(cytomegalovirus OR cytomegalic OR "cmv infection" OR "cmv infections" OR "ccmv infection" OR "ccmv infections" OR "cmv disease" OR "ccmv disease")	3,996
#2	(MH "Seroconversion") OR TI(seroconversion OR seroprevalence OR "sero-positive" OR seropositive OR seropositivity) OR AB(seroconversion OR seroprevalence OR "sero-positive" OR seropositive OR seropositivity)	6,996
#3	TI(congenital) OR AB(congenital)	28,402
#4	#1 AND #2 AND #3	48
#5	MH "Adolescence+" OR MH "Child+" OR MH "Pediatrics+" OR TI(Infant OR infants OR infancy OR newborn OR newborns OR neonatal OR neonate OR baby OR babies OR preterm OR toddler OR toddlers OR boy OR boys OR boyhood OR girl OR girls OR girlhood OR kid OR kids OR child OR children OR schoolchild OR school child OR school age OR adolescent OR adolescents OR adolescence OR juvenile OR juveniles OR youth OR youths OR teen OR teens OR teenager OR teenagers OR pubescent OR pubescence OR prepubescent OR prepubescence OR pediatric OR pediatrics OR paediatric OR paediatrics) OR AB(Infant OR infants OR infancy OR newborn OR newborns OR neonatal OR neonate OR baby OR babies OR preterm OR toddler OR toddlers OR boy OR boys OR boyhood OR girl OR girls OR girlhood OR kid OR kids OR child OR children OR schoolchild OR school child OR school age OR adolescent OR adolescents OR adolescence OR juvenile OR juveniles OR youth OR youths OR teen OR teens OR teenager OR teenagers OR pubescent	994,180

	OR pubescence OR prepubescent OR prepubescence OR pediatric OR pediatrics OR paediatric OR paediatrics)	
#6	#4 AND #5	45
#7	#6 NOT PT (Abstract OR Book OR Book Chapter OR Book Review OR Case Study OR Commentary OR Editorial OR Letter OR Masters Thesis OR Pamphlet OR Pamphlet Chapter OR Poetry)	43
#8	#7 AND LA English	43

Scopus

Search date: 10/9/2018

#1	TITLE-ABS-KEY (cytomegalovirus OR cytomegalic OR "cmv infection" OR "cmv infections" OR "ccmv infection" OR "ccmv infections" OR "cmv disease" OR "ccmv disease")	69,023
#2	TITLE-ABS-KEY (seroconversion OR seroprevalence OR "sero-positive" OR seropositive OR seropositivity)	94,190
#3	TITLE-ABS-KEY (congenital)	497,054
#4	#1 AND #2 AND #3	457
#5	TITLE-ABS-KEY (Infant OR infants OR infancy OR newborn OR newborns OR neonatal OR neonate OR baby OR babies OR preterm OR toddler OR toddlers OR boy OR boys OR boyhood OR girl OR girls OR girlhood OR kid OR kids OR child OR children OR schoolchild OR school child OR school age OR adolescent OR adolescents OR adolescence OR juvenile OR juveniles OR youth OR youths OR teen OR teens OR teenager OR teenagers OR pubescent OR pubescence OR prepubescent OR prepubescence OR pediatric OR pediatrics OR paediatric OR paediatrics)	1,735,772
#6	#4 AND #5	117
#7	#6 AND (LIMIT-TO (DOCTYPE , "ar") OR LIMIT-TO (DOCTYPE , "re"))	114
#8	#7 AND (LIMIT-TO (LANGUAGE , "English")	106

ClinicalTrials.gov

Search Date:

#1	(cytomegalovirus OR cytomegalic OR "cmv infection" OR "cmv infections" OR "ccmv infection" OR "ccmv infections" OR "cmv disease" OR "ccmv disease") AND (seroconversion OR seroprevalence OR "sero-positive" OR seropositive OR seropositivity)	56
#2	#1 Limit to Child birth-17	20
